# Serum TARC Levels in Patients with Systemic Sclerosis: Clinical Association with Interstitial Lung Disease

**DOI:** 10.3390/jcm10040660

**Published:** 2021-02-09

**Authors:** Ai Kuzumi, Ayumi Yoshizaki, Satoshi Ebata, Takemichi Fukasawa, Asako Yoshizaki-Ogawa, Yoshihide Asano, Koji Oba, Shinichi Sato

**Affiliations:** 1 Department of Dermatology, Graduate School of Medicine, The University of Tokyo, Tokyo 113-8655, Japan; KUZUMIA-DER@h.u-tokyo.ac.jp (A.K.); EBATAS-DER@h.u-tokyo.ac.jp (S.E.); FUKASAWAT-DER@h.u-tokyo.ac.jp (T.F.); YOSHIZAKIA-DER@h.u-tokyo.ac.jp (A.Y.-O.); ASANOY-DER@h.u-tokyo.ac.jp (Y.A.); SATOS-DER@h.u-tokyo.ac.jp (S.S.); 2 Department of Biostatistics, School of Public Health, Graduate School of Medicine, The University of Tokyo, Tokyo 113-8655, Japan; oba@epistat.m.u-tokyo.ac.jp

**Keywords:** systemic sclerosis, T helper 2, thymus and activation-regulated chemokine, interstitial lung disease, pulmonary function

## Abstract

Systemic sclerosis (SSc) is a multisystem fibrotic disorder with autoimmune background. Accumulating evidence has highlighted the importance of T helper (Th) 2 cells in the pathogenesis of SSc and its complications. Because thymus and activation-regulated chemokine (TARC) is a potent chemoattractant for Th2 cells, we measured serum TARC levels in SSc patients and analyzed their correlation with interstitial lung disease (ILD), a major complication of SSc. Serum TARC levels were significantly elevated in patients with SSc, especially in those with the diffuse subtype, compared with healthy controls. In particular, dcSSc patients with SSc-associated ILD (SSc-ILD) showed higher TARC levels than those without SSc-ILD. However, there was no significant correlation between serum TARC levels and pulmonary function in SSc patients. Serum TARC levels did not correlate with serum levels of interleukin-13, an important Th2 cytokine, either. Furthermore, in the longitudinal study, serum TARC levels did not predict the onset or progression of SSc-ILD in patients with SSc. These results were in contrast with those of KL-6 and surfactant protein D, which correlated well with the onset, severity, and progression of SSc-ILD. Overall, these results suggest that serum TARC levels are not suitable for monitoring the disease activity of SSc-ILD.

## 1. Introduction

Systemic sclerosis (SSc) is a progressive connective tissue disease in which excessive fibrosis of the skin and internal organs leads to diverse clinical manifestations that are highly distressing and often life-threatening [1,2]. Although the pathogenesis of SSc still remains unclear, various immunological abnormalities have been identified in SSc, indicating the autoimmune nature of the disease [3,4]. In particular, abnormalities of T cells have been extensively studied in both patients and animal models of SSc [5,6]. Notably, T-cell immune responses in SSc are heavily skewed toward T helper (Th) 2 with increased production of Th2 cytokines such as interleukin IL-4 and IL-13 [7,8,9]. Th2 cells drive the pathological cascade of SSc through multiple mechanisms [10]. Indeed, various cytokines and chemokines that are involved in activation, migration, and differentiation of Th2 cells have been implicated in the development of SSc [11,12,13,14,15].

Thymus and activation-regulated chemokine (TARC), also known as CC chemokine ligand 17, is a member of the CC chemokine group that is constitutively expressed in the thymus and is produced by keratinocytes, endothelial cells, bronchial epithelial cells, and other cell types [16]. TARC is a potent and selective chemoattractant for T cells expressing CC chemokine receptor 4 (CCR4) [17]. Because CCR4 is preferentially expressed on Th2 cells, TARC has been associated with Th2-dominant skin diseases, including atopic dermatitis, bullous pemphigoid, and cutaneous T-cell lymphoma [18,19,20]. For instance, TARC recruits CCR4^+^ Th2 cells into lesional skin in atopic dermatitis, where serum TARC levels are used as a sensitive biomarker for monitoring disease activity [21,22].

In recent years, TARC has also been implicated in interstitial lung disease (ILD), a common and severe complication of SSc. ILD is a group of inflammatory and fibrotic lung diseases with different etiologies such as infection, malignancy, drug-induced, and connective tissue diseases [23,24]. Yamane et al. have shown that serum TARC levels are elevated in patients with non-small cell lung cancer associated with ILD compared with patients without ILD [25]. They have also reported that TARC is a potential diagnostic marker for drug-induced ILD in the treatment of malignant lymphoma [26]. In the context of ILD caused by connective tissue diseases, serum TARC levels are increased in patients with dermatomyositis-associated ILD [27].

A previous study has demonstrated that serum TARC levels are elevated in SSc patients [28], which is in line with the Th2 dominance in SSc and the close association between TARC and Th2 immune responses. Accumulating evidence has shown that Th2 cells and Th2 cytokines are also critically involved in the development of SSc-associated ILD (SSc-ILD) [29,30]. However, there were no statistically significant differences in serum TARC levels between patients with SSc-ILD and those without SSc-ILD in the above study [28]. Due to the heterogenous and progressive nature of the disease, cytokine and chemokine profiles in SSc can vary by patient population, especially depending on the progression of SSc [31,32]. Therefore, to assess the involvement of TARC in SSc-ILD, it is important to examine TARC expression according to the severity and disease activity of SSc-ILD. However, there have been no studies investigating the association between serum TARC levels and the progression of SSc-ILD.

Here, to evaluate the relevance of TARC in SSc-ILD, we examined serum TARC levels in SSc patients and analyzed their correlations with clinical parameters in the longitudinal study.

## 2. Experimental Section

### 2.1. Patients

Serum samples were obtained from 74 Japanese SSc patients (36 women, 8 men; age [mean ± SD], 46.0 ± 16.2 years; disease duration, 1.46 ± 1.37 years) and 14 healthy controls (11 women, three men; age, 45.4 ± 10.8 years) at their first visit after getting written informed consent. All patients fulfilled the ACR/EULAR classification criteria for SSc [33]. Patients treated with corticosteroids or other immunosuppressants prior to their first visit were excluded. SSc patients were categorized by LeRoy’s classification system [34]: 55 diffuse cutaneous SSc (dcSSc) patients (age, 44.6 ± 16.6 years; disease duration, 1.30 ± 1.33 years) and 19 limited cutaneous SSc (lcSSc) patients (age, 48.9 ± 15.4 years; disease duration, 1.83 ± 1.47 years). In a retrospective longitudinal study, serum samples from 52 patients who had SSc-ILD at their first visit were obtained after the short-time follow-up period of 6.96 ± 1.60 months. The whole study was approved by the ethics committee of the University of Tokyo Graduate School of Medicine.

### 2.2. Clinical Assessment

Complete medical histories, physical examination, and laboratory tests were conducted on all patients at their first visit and at the end of the follow-up period. Disease onset was defined as the first clinical event of SSc other than Raynaud’s phenomenon. Disease duration was calculated as the interval between the onset of SSc and the time of blood sampling at their first visit. Skin thickness was measured by the modified Rodnan total skin thickness score as previously described [35]. The percentage of predicted forced vital capacity (%FVC) and the percentage of predicted diffusing capacity for carbon monoxide (%DLco) were measured by a pulmonary function test. Organ involvement was defined as follows: ILD = ground-glass opacity and/or reticular pattern on high-resolution computed tomography; pulmonary hypertension = right ventricular systolic pressure of 35 mmHg or higher on echocardiogram [36]; esophagus = gastroesophageal reflux disease detected at upper gastrointestinal endoscopy; kidneys = malignant hypertension and/or rapidly progressive renal failure.

### 2.3. Measurement of Serum TARC and IL-13 Levels

Serum TARC and IL-13 levels were evaluated using enzyme-linked immunosorbent assay kits (R&D Systems, Minneapolis, MN, USA). Briefly, microplates coated with anti-TARC antibodies or anti-IL-13 antibodies were incubated with serum for 2 h (for TARC) or 1 h (for IL-13). Subsequently, the wells were washed and incubated for 1 h (for TARC) or 30 min (for IL-13) with horseradish peroxidase-conjugated antibodies against TARC or IL-13. Then, the wells were washed again, supplemented with tetramethylbenzidine, and incubated for 30 min. Finally, sulfuric acid was added to terminate the reaction. The absorbance at 450 nm was measured. Serum TARC and IL-13 levels were calculated from a standard curve.

### 2.4. Statistical Analysis

Data are presented as means ± SD. Statistical analysis was performed by Mann-Whitney U-test for two-group comparison, Fisher’s exact probability test for frequency analysis, and Spearman’s rank correlation coefficient for clinical correlations. P values less than 0.05 were considered statistically significant. All analyses were performed using GraphPad Prism 7.03 (GraphPad Software, La Jolla, CA, USA).

## 3. Results

### 3.1. Serum TARC Levels in Patients with SSc

**Figure 1:**
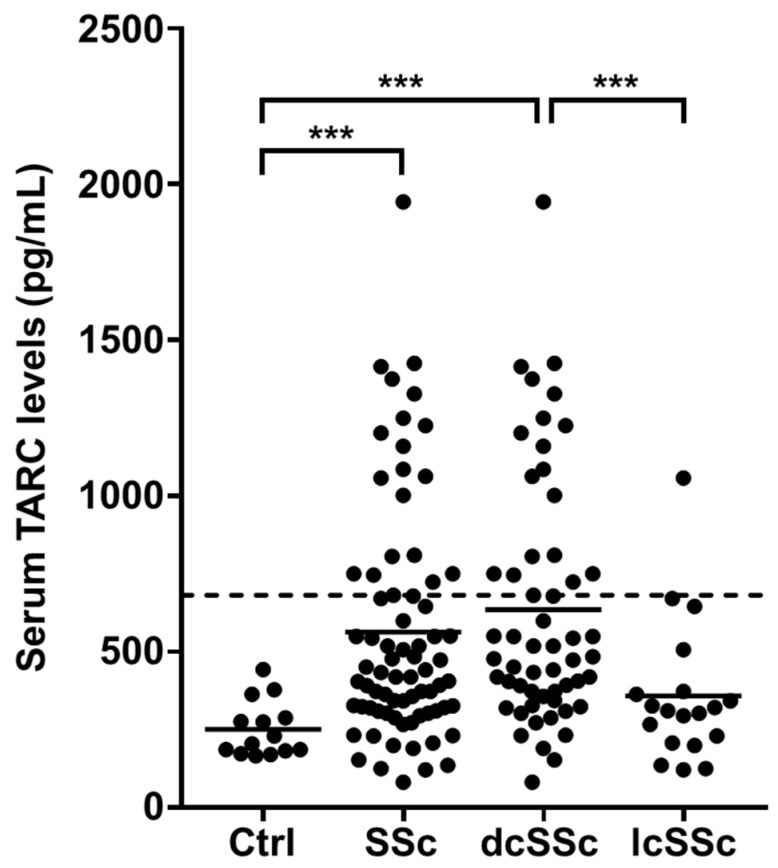
Serum TARC levels in patients with systemic sclerosis (SSc)**.** Serum TARC levels were determined in diffuse cutaneous systemic sclerosis (dcSSc) patients (*n* = 55), limited cutaneous systemic sclerosis (lcSSc) patients (*n* = 19), and healthy controls (Ctrl; *n* = 14) by a specific enzyme-linked immunosorbent assay kit. The horizontal lines represent the mean values. The broken line represents the cut-off value (mean + 2SD of the healthy controls). Mann-Whitney U-test was performed for two-group comparison. *** *p* < 0.001.

Initially, we measured serum TARC levels in 74 SSc patients and 14 healthy controls at their first visit. Serum TARC levels were significantly elevated in SSc patients compared to the healthy controls (563.4 ± 381.4 pg/mL vs. 251.3 ± 89.4 pg/mL, *p* < 0.0001; Figure 1). Within the SSc subgroups, dcSSc patients showed higher TARC levels (634.5 ± 399.1 pg/mL) than the healthy controls (*p* < 0.0001), while there were no statistically significant differences in TARC levels between the lcSSc patients (357.6. ± 277.8 pg/mL) and the healthy controls (*p =* 0.14).

### 3.2. Clinical Association of Serum TARC Levels in SSc Patients

**Figure 2:**
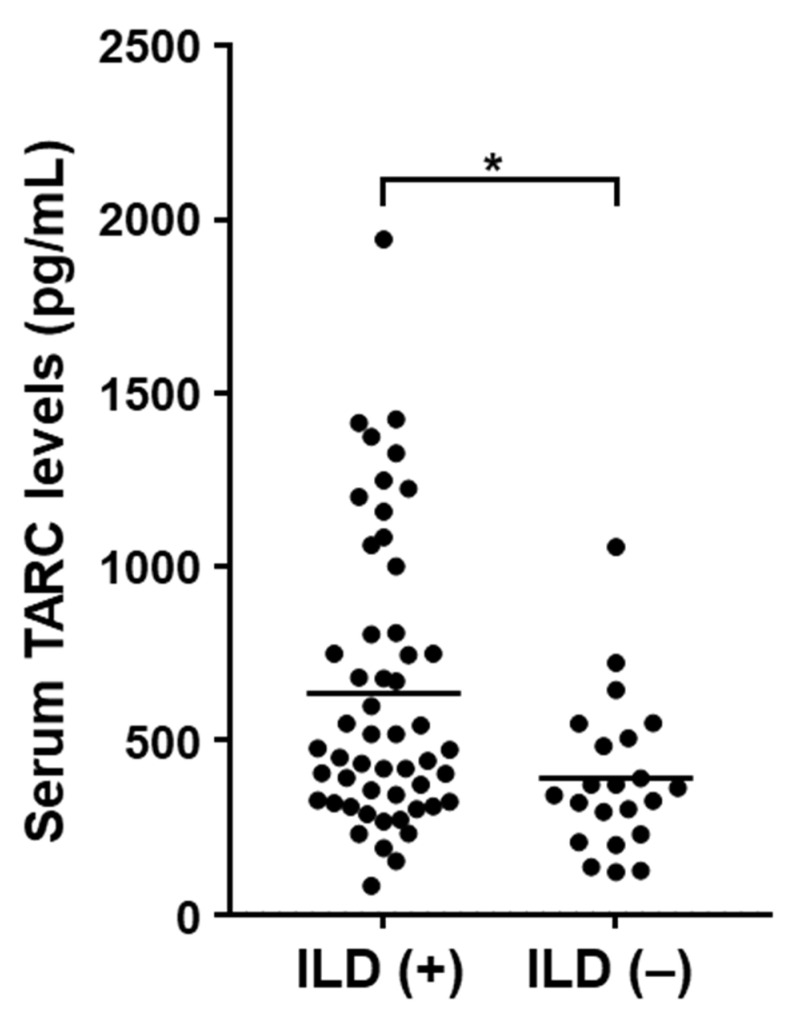
Serum TARC levels in SSc patients with or without SSc-ILD. Serum TARC levels were compared between SSc patients with and without SSc-ILD. Mann-Whitney U-test was performed for two-group comparison. * *p* < 0.05.

Next, clinical features were compared between SSc patients with elevated serum TARC levels and those with normal levels (Table 1). Values more than the mean + 2 SD (681.3 pg/mL) of the control serum samples were considered elevated. Serum TARC levels were increased in 27.0% (20/74) of SSc patients. Within the SSc subgroups, TARC levels were increased in 34.5% (19/55) of dcSSc patients and 5.2% (1/19) of lcSSc patients. There were no significant differences in sex, age, disease duration, or autoantibody type between patients with elevated TARC levels and those with normal levels. Of note, patients with elevated TARC levels were more frequently categorized as dcSSc than those with normal levels (95.0% vs. 66.7%, *p =* 0.015). Furthermore, patients with elevated TARC levels were more frequently accompanied by SSc-ILD (90.0% vs. 63.0%, *p =* 0.025). Consistent with this finding, serum TARC levels in SSc patients with ILD were significantly higher compared with those without ILD (636.1 ± 412.3 pg/mL vs. 391.7 ± 221.0 pg/mL, *p =* 0.010; Figure 2). However, there were no significant differences in %FVC or %DLco between patients with elevated TARC levels and those with normal levels (*p =* 0.504 for %FVC and *p =* 0.299 for %DLco). Within the SSc subtype, elevated serum TARC levels were associated with SSc-ILD in dcSSc but not in lcSSc patients. Pulmonary hypertension was not associated with the elevated serum TARC levels in either dcSSc or lcSSc patients (Tables S1 and S2). We found no significant differences in other clinical features between SSc patients with elevated and normal TARC levels.

### 3.3. Correlation between Serum TARC Levels and the Severity of SSc-ILD

**Figure 3:**
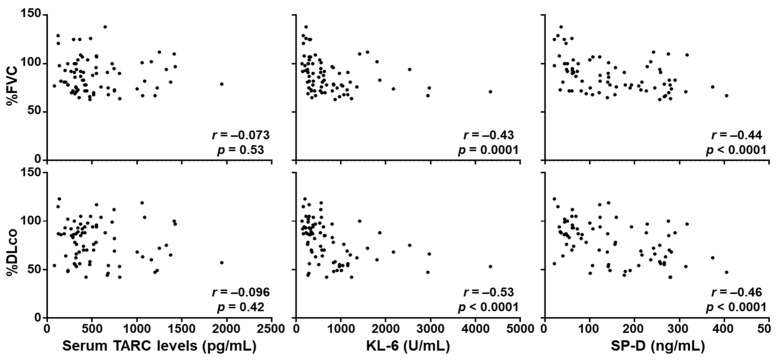
Correlations of serum TARC, KL-6, and SP-D levels with pulmonary function. There were no significant correlations between serum TARC levels and %FVC or % DLco. Serum KL-6 and SP-D levels were significantly negatively correlated with %FVC and %DLco. Correlations were assessed by Spearman’s rank correlation test.

Subsequently, we analyzed the correlation between serum TARC levels and pulmonary function in the whole SSc patients. Serum TARC levels did not correlate with %FVC (*r* = −0.0733, *p* = 0.535) or %DLco (*r* = −0.0959, *p* = 0.416; Figure 3). We also examined the correlation of serum levels of KL-6 and surfactant protein-D (SP-D), the conventional biomarker for SSc-ILD [37,38,39,40,41], with pulmonary function. Both KL-6 and SP-D showed significant negative correlation with %FVC (*r =* −0.430, *p =* 0.0001 for KL-6, *r =* −0.531, *p* < 0.0001 for SP-D) and %DLco (*r =* −0.438, *p* < 0.0001 for KL-6, *r = −*0.456, *p* < 0.0001 for SP-D). These results suggest that serum TARC levels are not associated with the severity of SSc-ILD. Because Th2 immune responses has been implicated in SSc-ILD [29,30], we also measured serum IL-13 levels in the same SSc patient group. However, these was no significant correlation between serum levels of TARC and IL-13 in SSc patients (*r =* 0.153, *p =* 0.192 Figure S1). Together with the lack of correlation between serum TARC levels and the severity of SSc-ILD, this result suggests that serum TARC levels in SSc patients might not necessarily reflect Th2 immune responses nor their contribution to SSc-ILD.

### 3.4. Correlation between Serum TARC Levels at the First Visit and the Development of SSc-ILD during the Follow-up Period

**Figure 4:**
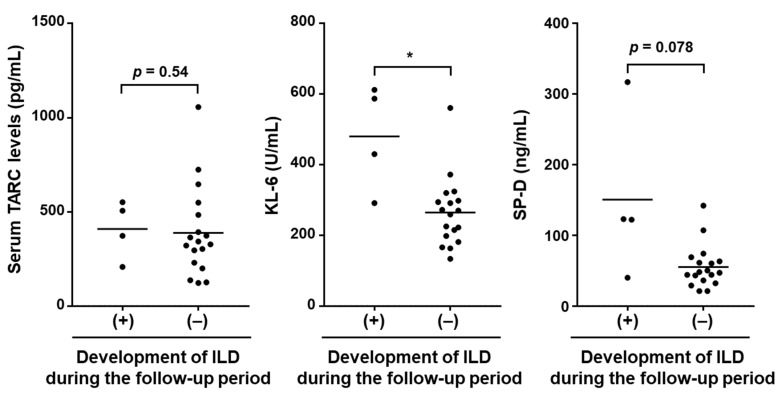
Correlations of serum levels of TARC, KL-6, and SP-D at the first visit with the development of SSc-ILD during the follow-up period. Serum levels of TARC, KL-6, and SP-D levels were analyzed in SSc patients without ILD at their first visit (*n* = 22) and were compared between patients who developed SSc-ILD and those who did not develop SSc-ILD during the follow-up period. Mann-Whitney U-test was performed for two-group comparison. * *p* < 0.05.

Next, we investigated whether serum TARC levels in SSc patients at their first visit would predict the newly onset of SSc-ILD during the follow-up period (6.96 ± 1.60 months). For this purpose, 22 SSc patients who did not have SSc-ILD at their first visit were enrolled in the analysis. During the follow-up period, four patients developed SSc-ILD. As shown in Figure 4, there were no significant differences in serum TARC levels between SSc patients who newly developed SSc-ILD during the follow-up period and those who did not (408.7 ± 154.6 pg/mL vs. 388.0 ± 236.7 pg/mL, *p =* 0.538). On the other hand, serum KL-6 levels were significantly higher in SSc patients who developed SSc-ILD during the follow-up period compared with those who did not (480.0 ± 149.5 pg/mL vs. 264.7 ± 98.04 pg/mL, *p =* 0.0011). Similar trends were obtained with serum SP-D levels, but the difference was not statistically significant (150.5 ± 117.6 pg/mL vs. 55.06 ± 30.06, *p =* 0.078). These results suggest that serum TARC levels cannot be used to predict the onset of SSc-ILD.

### 3.5. Correlation of Serum TARC Levels at the First Visit and the Progression of SSc-ILD during the Follow-up Period

**Figure 5:**
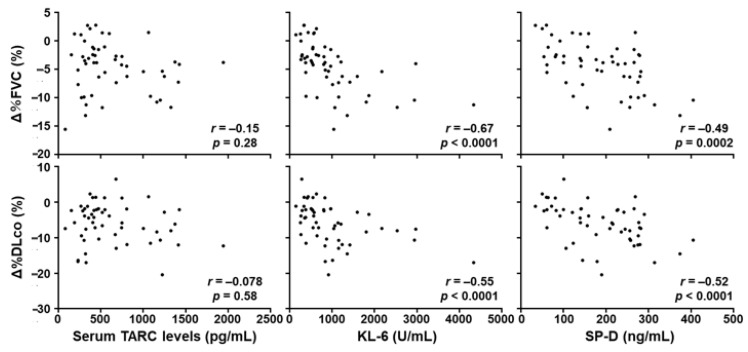
Correlations of serum levels of TARC, KL-6, and SP-D at the first visit with the changes of pulmonary function during the follow-up period. Serum levels of TARC, KL-6, and SP-D levels were analyzed in SSc patients with ILD at their first visit (*n* = 52), and their correlations with the changes of %FVC and %DLco after the follow-up period were evaluated. Correlations were assessed by Spearman’s rank correlation test.

We further examined whether serum TARC levels in SSc patients at their first visit would predict the progression of SSc-ILD during the follow-up period (Figure 5). For this purpose, 52 SSc patients who had SSc-ILD at their first visit were enrolled in the analysis. There were no significant correlations between serum TARC levels at their first visit and the change of %FVC or %DLco during the follow-up period (*r* = −0.151, *p =* 0.285 for %FVC, *r =* 0.0777, *p =* 0.584 for %DLco). In contrast, there were significant negative correlations between serum KL-6 and SP-D levels at their first visit and the change of %FVC (*r =* −0.672, *p* < 0.0001 for KL-6, *r =* −0.491, *p =* 0.0002 for SP-D) and %DLco (*r = −*0.545, *p* < 0.0001 for KL-6, *r =* −0.523, *p* < 0.0001 for SP-D) during the follow-up period. These results suggest that serum TARC levels are not a valid predictor for the short-time progression of SSc-ILD.

### 3.6. Correlation between the Change of Serum TARC Levels and the Progression of SSc-ILD during the Follow-up Period

**Figure 6:**
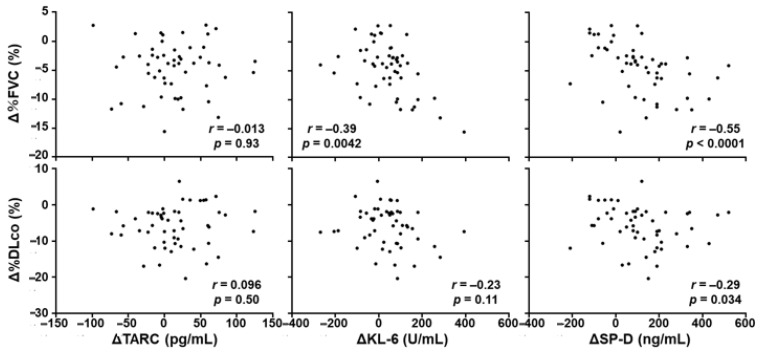
Correlations between the changes of serum TARC, KL-6, and SP-D levels and the changes of pulmonary function during the follow-up period. Correlations between the changes of serum TARC, KL-6, and SP-D levels and the changes of %FVC and %DLco during the follow-up period in SSc patients with SSc-ILD at their first visit (*n* = 52). Correlations were assessed by Spearman’s rank correlation test.

We also investigated whether the change of serum TARC levels in SSc patients would be correlated with the change of %FVC and %DLco during the follow-up period (Figure 6). For this purpose, 52 SSc patients who had SSc-ILD at their first visit were enrolled in the analysis. There were no significant correlations between the change of serum TARC levels and the change of %FVC or %DLco during the follow-up period (*r =* −0.0132, *p =* 0.926 for %FVC, *r =* 0.0964, *p =* 0.497). In contrast, there were significant negative correlation between the change of serum KL-6 and SP-D levels, and the change of %FVC (*r = −*0.391, *p =* 0.0042 for KL-6, *r =* −0.546, *p* < 0.0001 for SP-D). Similarly, the change of serum KL-6 and SP-D levels negatively correlated with the change of %DLco (*r =* −0.225, *p =* 0.109 for KL-6, *r =* −0.294, *p =* 0.034 for SP-D), although the correlation was not statistically significant between the change of KL-6 and %DLco. These results suggest that serum TARC levels cannot be used to monitor the disease activity of SSc-ILD.

## 4. Discussion

In this study, we demonstrated that serum TARC levels were significantly elevated in patients with dcSSc compared with healthy controls. Of note, serum TARC levels were significantly higher in SSc patients with ILD than those without ILD. However, there were no significant correlations between serum TARC levels and %FVC or %DLco in SSc patients. In the longitudinal analysis, serum TARC levels at the first visit did not predict the onset or progression of SSc-ILD during the short-term follow-up period. Changes of serum TARC levels did not correlate with the changes of %FVC or %DLco, either. In contrast, KL-6 and SP-D, the conventional biomarkers for ILD, were well correlated with the onset, severity, and disease activity of SSc-ILD in the same patient population. These results suggest that although elevated serum TARC levels are associated with the presence of SSc-ILD, TARC levels do not correlate with the severity of SSc-ILD, suggesting that TARC cannot be a predictor of fibrotic lesions.

The present study showed that elevated serum TARC levels were associated with higher prevalence of SSc-ILD in patients with SSc. In a previous study, SSc patients with elevated serum TARC levels tended to be more frequently accompanied by SSc-ILD compared with those with normal levels, but it did not reach a statistical significance [28]. The reason for this discrepancy is unclear but may be attributed to the difference in patient population: the overall prevalence of ILD among SSc patients in the previous study was low compared with our study (up to 42% vs. 70%). In addition, the previous study included SSc patients treated with steroids, whereas this study did not. Taken together, our results suggest that elevated serum TARC levels in SSc patients are, at least to some extent, associated with the presence of SSc-ILD.

In SSc, Th2 cells and Th2 cytokines contribute to excessive extracellular matrix deposition by promoting activation and collagen production of fibroblasts both directly and indirectly [10]. A recent study has demonstrated that T cell differentiation was significantly skewed toward Th2 in patients with SSc-ILD [29]. In line with this, CC chemokine 2, also known as monocyte chemoattractant protein-1, a chemokine promoting Th2 cell differentiation as well as migration of T cells and monocytes, has emerged as a potential biomarker for SSc-ILD. Serum levels of CC chemokine 2 are significantly correlated with the presence and severity of SSc-ILD and also predict the decline of pulmonary function in SSc patients [14]. Although TARC is also critically involved in Th2 immune responses by promoting the migration of Th2 cells, serum TARC levels did not correlate with the onset, severity, or progression of SSc-ILD. Therefore, serum TARC level might not necessarily reflect the pathophysiological process of SSc-ILD, including Th2 immune responses. Indeed, serum TARC levels did not correlate with serum IL-13 levels in SSc patients. However, the pathophysiological process of SSc-ILD is complicated and not limited to Th2 immune responses. For instance, SSc-ILD is associated with IL-8, a neutrophil chemoattractant; CXCL10, a chemoattractant for various immune cells; and CXCL11, a chemoattractant for activated T cells [42,43,44,45,46]. Therefore, evaluating the correlation between serum levels of TARC and these chemokines in SSc patients, especially in dcSSc patients with SSc-ILD, might provide a broader insight into the possible implications of TARC in SSc-ILD. Although further mechanistic studies such as stimulating fibroblasts with TARC are needed to investigate whether and to what extent TARC is involved in SSc-ILD, serum TARC levels do not predict the severity or disease activity of SSc-ILD, which suggests that TARC does not directly cause fibrosis.

In summary, this study examined the serum TARC levels in SSc patients and evaluated their correlation with SSc-ILD. Although elevated serum TARC levels were associated with the presence of SSc-ILD in patients with SSc, serum TARC levels did not correlate with the onset, severity, or progression of SSc-ILD. Although preliminary, this study shows that serum TARC level is not a predictor for assessing the severity or disease activity of SSc-ILD.

## 5. Conclusions

Our data indicate that serum TARC levels were higher in patients with dcSSc compared with healthy controls. In particular, serum TARC levels were elevated in dcSSc patients with SSc-ILD than those without SSc-ILD. However, serum TARC levels did not correlate with the severity or disease activity of SSc-ILD. Furthermore, serum TARC levels cannot predict the onset or the exacerbation of the disease. These results suggest that serum TARC level is not a predictor of disease activity in SSc-ILD, which implies TARC is not necessarily the cause of fibrotic lesions.

## Figures and Tables

**Table 1:** Clinical and laboratory features of SSc patients.

Clinical and Laboratory Features	Serum TARC Levels		
	Elevated (*n* = 20)	Normal (*n* = 54)	*p* values
Sex, number of women/men	18/2	48/6	>0.999
Age, mean years ± SD	51.6 ± 15.5	48.9 ± 17.0	0.601
Disease duration, mean years ± SD	3.0 ± 2.7	3.4 ± 4.7	0.647
Disease pattern, No. of dcSSc/lcSSc	19/1	36/18	0.015 *
MRSS, mean points ± SD	12.5 ± 7.7	11.5 ± 8.2	0.480
Autoantibodies			
Anti-topoisomerase I	75% (15/20)	80% (43/54)	0.753
Anticentromere	10% (2/20)	30% (16/54)	0.126
Anti-RNA polymerase III	5% (1/20)	9% (5/54)	>0.999
Organ involvement			
Lung			
ILD	90% (18/20)	63% (34/54)	0.025 *
FVC, mean % predicted ± SD	85.2 ± 15.3	89.3 ± 18.3	0.504
DLco, mean % predicted ± SD	74.4 ± 23.2	80.0 ± 20.0	0.299
Pulmonary hypertension	0% (0/20)	7% (4/54)	0.569
Esophagus	85% (17/20)	69% (38/54)	0.245
Kidneys	5% (1/20)	2% (1/54)	0.470
Cutaneous symptoms			
Raynaud’s phenomenon	95% (19/20)	91% (49/54)	>0.999
Nail fold bleeding	60% (12/20)	61% (33/54)	>0.999
Telangiectasia	30% (6/20)	56% (30/54)	0.068
Pitting scars	45% (9/20)	50% (27/54)	0.796
Digital ulcers	25% (5/20)	39% (21/54)	0.411

Unless noted otherwise, values are percentages and values in parentheses represent the number of patients. dcSSc, diffuse cutaneous systemic sclerosis; lcSSc, limited cutaneous systemic sclerosis; MRSS, modified Rodnan total skin thickness score; ILD, interstitial lung disease; FVC, forced vital capacity; DLco, diffusing capacity for carbon monoxide. * *p* < 0.05 vs. SSc patients with normal serum TARC levels.

## Data Availability

The data presented in this study are available on request from the corresponding author. The data are not publicly available due to ethical reasons.

## Supplementary Materials

The following are available online at https://www.mdpi.com/2077-0383/10/4/660/s1, Figure S1: Correlation of serum TARC levels with serum IL-13 levels. Table S1: Clinical features of dcSSc patients, Table S2: Clinical features of lcSSc patients.
