# Supplementary material for: Serum TARC Levels in Patients with Systemic Sclerosis: Clinical Association with Interstitial Lung Disease
Source: J Clin Med. 2021 Feb 9;10(4):660. doi: 10.3390/jcm10040660 (PMC7915627; doi:10.3390/jcm10040660)
Supplement: Supplementary file 1 [file jcm-10-00660-s001.pdf]

Supplementary Data

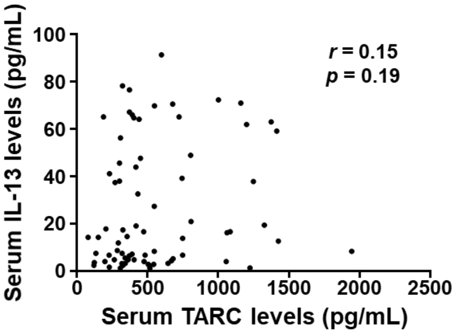


**Figure 1.** Correlation of serum TARC levels with serum IL-13 levels.

There was no significant correlation between serum levels of TARC and IL-13 in SSc patients. Correlation was assessed by Spearman's rank correlation test.

**Table 1.** Clinical features of dcSSc patients.

| **Clinical and Laboratory Features** | **Serum TARC Levels** | | |
|---|---|---|---|
| | Elevated (*n* = 19) | Normal (*n* = 36) | *p* values |
| ILD | 95% (18/19) | 69% (25/36) | 0.041* |
| Pulmonary hypertension | 0% (0/20) | 3% (1/36) | >0.999 |

Values are percentages and values in parentheses represent the number of patients. *$p < 0.05$ vs. dcSSc patients with normal serum TARC levels.

**Table 2.** Clinical features of lcSSc patients.

| **Clinical and Laboratory Features** | **Serum TARC Levels** | | |
|---|---|---|---|
| | Elevated (*n* = 1) | Normal (*n* = 18) | *p* values |
| ILD | 0% (0/1) | 50% (9/18) | >0.999 |
| Pulmonary hypertension | 0% (0/1) | 11% (2/18) | >0.999 |

Values are percentages and values in parentheses represent the number of patients.
